# Modern Approach to Diabetic Retinopathy Diagnostics

**DOI:** 10.3390/diagnostics14171846

**Published:** 2024-08-24

**Authors:** Maria Kąpa, Iga Koryciarz, Natalia Kustosik, Piotr Jurowski, Zofia Pniakowska

**Affiliations:** 1 Department of Ophthalmology and Vision Rehabilitation, Medical University of Lodz, 90-549 Lodz, Poland; maria.kapa@stud.umed.lodz.pl (M.K.); natalia.kustosik@stud.umed.lodz.pl (N.K.); piotr.jurowski@umed.lodz.pl (P.J.); zofia.pniakowska@umed.lodz.pl (Z.P.); 2 Optegra Eye Clinic, 90-127 Lodz, Poland

**Keywords:** diabetes, diabetic retinopathy, neovascularization, retinal nonperfusion, ultra-widefield fluorescein angiography, diagnostics, ophthalmology, artificial intelligence, telemedicine

## Abstract

This article reviews innovative diagnostic approaches for diabetic retinopathy as the prevalence of diabetes mellitus and its complications continue to escalate. Novel techniques focus on early disease detection. Technological innovations, such as teleophthalmology, smartphone-based photography, artificial intelligence with deep learning, or widefield photography, can enhance diagnostic accuracy and accelerate the treatment. The review highlights teleophthalmology and handheld photography as promising solutions for remote eye care. These methods revolutionize diabetic retinopathy screening, offering cost-effective and accessible solutions. However, the use of these techniques may be limited by insurance coverage in certain world regions. Ultra-widefield photography offers a comprehensive view of up to 80.0% of the retina in a single image, compared to the 34.0% coverage of the traditional seven-field imaging protocol. It allows retinal imaging without pupil dilation, especially for individuals with compromised mydriasis. However, they also have drawbacks, including high costs, artifacts from eyelashes, eyelid margins, and peripheral distortion. Recent advances in artificial intelligence and machine learning, particularly through convolutional neural networks, are revolutionizing diabetic retinopathy diagnostics, enhancing screening efficiency and accuracy. FDA-approved Artificial Intelligence-powered devices such as LumineticsCore™, EyeArt, and AEYE Diagnostic Screening demonstrate high sensitivity and specificity in diabetic retinopathy detection. While Artificial Intelligence offers the potential to improve patient outcomes and reduce treatment costs, challenges such as dataset biases, high initial costs, and cybersecurity risks must be considered to ensure safety and efficiency. Nanotechnology advancements further enhance diagnosis, offering highly branched polyethyleneimine particles with fluorescein sodium (PEI-NHAc-FS) for better fluorescein angiography or vanadium oxide-based metabolic fingerprinting for early detection.

## 1. Introduction

Diabetes mellitus (DM) is one of the most common hormonal disorders worldwide and a major health challenge in recent times [1]. With a notable increase in prevalence, there is an urgent requirement to seek effective management.

DM is associated with a multitude of complications, including heart disease, strokes, and renal and ocular damage, including retinopathy and nerve damage [2].

The increasing occurrence of diabetes is associated with the aging population. The 10th edition of the *IDF Diabetes Atlas* offers updated estimates and projections of DM prevalence, as well as health expenditure related to diabetes, on national, regional, and global scales. More than half a billion people worldwide are currently living with DM, which amounts to over 10.5% of the global adult population [3]. If this tendency continues, there will be a rise in people with DM to 12.2% (783.2 million) in 2045 [3].

Future projections indicate that by the year 2045, the absolute number of individuals with diabetes will have risen by 46% [4], emphasizing the seriousness of the problem. Furthermore, there will be an increasing number of patients with DM complications, including diabetic retinopathy, which we would like to focus on in this review. Diabetic retinopathy (DR) is a microvascular disease affecting approximately one-third of DM patients, with severe indications, such as proliferative diabetic retinopathy (PDR) or diabetic macular edema (DME), possibly leading to irreversible vision loss [5].

Optimal control of blood glucose, lipid profile, and arterial blood pressure are essential for reducing the risk of development and progression. These measures help mitigate the microvascular damage and inflammatory process [6].

A recent study based on data from nationally representative and local population-based studies estimated that in 2021, approximately 9.60 million individuals were living with DR, with a prevalence rate of 26.43% among people with diabetes [7]. Additionally, 1.84 million individuals were living with vision-threatening DR, corresponding to a prevalence rate of 5.06% among people with diabetes [7].

In this article, we will focus on innovative approaches to the diagnosis and treatment of DR.

## 2. Advancements in Diagnosing Diabetic Retinopathy

To reduce preventable vision loss in DR, current approaches emphasize early intervention and disease management. Technological advancements, such as teleophthalmology, smartphone-based photography (SBP), and artificial intelligence (AI) with deep learning (DL), are reshaping diagnostic trends. Additionally, imaging techniques like Colour Fundus Photography (CFP), Fundus Fluorescein Angiography (FFA), Optical Coherence Tomography (OCT), and OCT Angiography (OCTA) are evolving toward widefield photography (WFP). Slit-Lamp Biomicroscopy (SLB) continues to play a crucial role in referral and treatment decisions; however, given the advancements in imaging modalities, it is advisable to integrate these new technologies into clinical practice [8].

The escalating prevalence of diabetes and its associated complications, particularly DR, alongside the imperative for routine screenings, poses a significant challenge for healthcare systems worldwide [9]. Some developed nations, such as the United Kingdom, have successfully implemented national screening programs for DR, effectively reducing complications and preventing blindness within local communities; such initiatives are rare on a global scale due to their considerable costs [10].

### 2.1. Teleophthalmology

Teleophthalmology emerges as a cost-effective solution, leveraging advanced imaging and technology to provide healthcare services remotely [11]. Research corroborates its efficacy in remote consultations, demonstrating high sensitivity and specificity for detecting retinopathy and effectively triaging patients based on disease screening grade severity, empowering ophthalmologists to focus more on therapeutic interventions [12]. For instance, in a recent three-year study involving 1646 patients, teleophthalmology demonstrated promising results in grading retinal images for DR, achieving an average sensitivity of 90.0% and specificity of 84.6% [13]. The authors emphasized that accuracy showed continuous improvement over time, attributed to training on the website platform and long-term practice. These findings establish telemedicine as a crucial tool in delivering comprehensive eye care [14]. Although teleophthalmology has been shown to improve adherence to follow-up recommendations, in countries like the USA where telehealth services are not extensively covered by insurance, a study by Lieng et al. revealed that despite the established benefits of remote retinal imaging, the absence of immediate access to teleophthalmology services limits its efficacy in enhancing access to eye care [15].

### 2.2. Handheld Photography

One significant development in this field is the advent of high-quality smartphone cameras, which have facilitated the creation of smartphone-based fundus photography (SBFP), employing a handheld condensing lens and a smartphone camera to capture images of the retina. 

This method, together with other handheld devices, has proven to be an invaluable clinical tool, particularly in regions with geographical or financial limitations [16]. A study by de Oliveira et al. compared a handheld fundus camera the Eyer (Phelcom Technologies, LLC, Massachusetts, USA) with standard tabletop cameras for DR and DME screening, showing high agreement between the devices and similar image quality. Importantly, the Eyer was significantly more affordable, costing approximately USD 4500 USD compared to USD 25,000 USD for tabletop cameras in Brazil in March 2023 [15].

The convenience of handheld photography, which can be operated by non-ophthalmic personnel and utilized outside of ophthalmic clinics, coupled with its integration with artificial intelligence for rapid image analysis, greatly improves accessibility [17]. Consequently, it facilitates the early detection of diseases. In addition, the Remidio Vistaro smartphone device (Remidio Innovative Solutions Pvt. Ltd., Bangalore, India), which offers mydriatic imaging with autofocus, has gained prominence in widefield retinal imaging. It captures high-quality images (91.6% clinically useful) with a wide field of view (65°) within 15 s. Its two-field montage surpasses the seven-field Early Treatment DR Study image, enabling efficient screening for retinal pathologies [18]. A recent evaluation showed both nonmydriatic ultra-widefield imaging (UWFI) and mydriatic widefield imaging (WFI) using Vistaro (Remidio Innovative Solutions Pvt. Ltd., Bangalore, India) had high sensitivity (96.6%) and specificity (92.7%) in detecting sight-threatening DR, with UWFI identifying more peripheral DR lesions (50.3% vs. 27.9%) [19].

A recent study by Malerbi et al. assessed the efficacy of AI systems integrated into a portable handheld retinal camera (Eyer) for the detection of DR, and more-than-mild DR (mtmDR) presented notable sensitivity and specificity rates [20]. Specifically, the AI system (Diabetic Retinopathy Alteration Score, DRAS; Retinal Alteration Score RAS), demonstrated a sensitivity of 90.5% and a specificity of 90.7% for the detection of DR, while for mtmDR, the sensitivity and specificity were recorded as 90.2% and 85.1%, respectively. Their results correspond with other values mentioned in the literature regarding the detection of DR with portable devices, which are summarized in Table 1. 

### 2.3. Widefield and Ultra-Widefield Imaging

For the last three decades, the seven-field imaging protocol, capturing two posterior and five mid-peripheral fields, has been the gold standard in DR clinical trials. However, it covers only 34.0% of the retina and requires significant patient cooperation, stable fixation, and time [34,35]. Ultra-wide field photography is a significant advancement, offering a comprehensive view of the retina, extending to previously inaccessible peripheral areas. This technology captures up to 80.0% or nearly 200° of the retina in a single image, which is crucial for detecting and classifying DR, as manifestations often occur in the retinal periphery [36]. Silva et al. found undetected peripheral lesions associated with a 4.7 times higher risk of DR progression and PDR development within four years [37]. Furthermore, it allows for retinal imaging without pupil dilatation, enhancing convenience and speed, especially for individuals with compromised mydriasis [38,39].

In a comparative study conducted by Duncan et al., the Ultra-Widefield Color (UWF-C) cameras Clarus (Clarus 500 and 700: version 1.0.2 or higher (Carl Zeiss Meditec, Jena, Germany) and Optos (Vantage 3.4, Advance 4.2, or higher, Optos PLC, Dunfermline, Scotland, United Kingdom); showed good agreement with the Standard Seven-Field system for DR detection and staging severity. However, the UWF-C systems had a smaller proportion of ungradable images, 2.1% and 1.0% for Clarus and Optos, respectively, compared to 5.2% for 7F imaging. Furthermore, the use of UWF-C cameras to visualize areas outside the 7F region enabled the detection of proliferative lesions, which elevated the DR severity level [35]. Moreover, studies by Talks et al. and Wessel et al., which used UWFI, detected retinal neovascularisation in 11.7% and 17% of PDR cases, respectively, which were missed by standard seven-field imaging as they were located outside its coverage area [40,41], highlighting the importance of this modern approach. 

Although UWF-C cameras appear promising, they are not without their drawbacks, including high cost, artifacts from eyelashes, eyelid margin, nose, and peripheral distortion [17]. Rajalakshmi et al. noted that UWF cameras in their studies had color variations in retinal images due to different laser wavelengths, making it challenging to identify certain peripheral lesions, such as intraretinal microvascular abnormalities [17]. Similarly, Srinivasan et al. found a 15.2% disagreement between observers in grading DR on UWF fundus, attributed to low resolution for detecting small retinal lesions, especially microaneurysms. This disagreement underlines the importance of consideration when using UWF systems in clinical studies [42].

In this context, the introduction of Color Red–Green–Blue (RGB) technology in retinal imaging represents a significant advancement. By using simultaneous red, green, and blue laser scans, this technology generates natural color images of the retina, offering a more accurate and detailed view compared to the earlier Red–Green (RG) system, which relies solely on red and green lasers. This enhanced imaging capability is particularly valuable for the clinical identification and assessment of vitreoretinal, retinal, and chorioretinal pathologies [43,44].

Among the available options discussed in Table 2, UWFI systems such as the Optos Optomap and the Clarus are the most commonly used. Xiao et al. found that the Clarus system, with its high-resolution red, green, and blue channels, offers superior detection of subtle DR lesions. The Clarus system is noted for its accuracy and fewer artifacts, demonstrating greater severity of DR and excelling in early detection compared to both the five-field and Optos systems [45].

### 2.4. UWF Fluorescein Angiography, Optical Coherence Tomography, and OCT Angiography

Current progress in technology facilitates the implementation of UWF fluorescein angiography (FA), OCT, and OCTA, enabling users to overcome limited field of view (FOV), which can contribute to inaccurate assessment of the severity of DR.

FA is the gold standard for advanced DR evaluation and detecting retinal neovascularisation (NV) [46]. Integrating UWF imaging with FA improves diagnostic efficiency and pathology detection. However, due to potential side effects, OCTA gains recognition for non-invasive assessment of diabetic microvascular features, including NV. Studies using widefield OCTA (WF-OCTA) revealed a 95.0% sensitivity in PDR detection. WF-OCTA detected 93.0% of NV elsewhere (NVE) in the temporal quadrants, where they are more prevalent. Additionally, 72.0% of all NVEs visible on UWF-FA were detected within the field of view using WF-OCTA [40,47]. These results are comparable with other studies, highlighting the efficacy of WF-OCTA in PDR diagnosis and DR assessment [48,49,50].

Advancements in OCTA technology have significantly improved imaging capabilities, with the ability to capture image areas of 24 × 20 mm² (approximately 120° FOV), representing a substantial upgrade over previous systems. Comparing the scanning areas of 24 mm × 20 mm with 12 mm × 12 mm, the high-speed ultra-widefield swept-source OCTA (SS-OCTA) offers a more accurate assessment of retinal ischemia and detects a greater number of NVs and intraretinal microvascular abnormalities (IRMAs) [51]. Zhao et al. employed a novel ultra-widefield swept-source OCTA (UWF-SS-OCTA) using BM-400 K (BMizar, TowardPi Medical Technology Co., Ltd., Beijing, China) to investigate retinal and choroidal alterations in diabetic patients without clinical DR (DM-NoDR) [52]. Their study unveiled early microvascular impairments, such as non-perfusion areas (NPA) and capillary tortuosity, in both central and peripheral regions of DM-NoDR eyes. Notably, UWF-SS-OCTA detected undetectable microaneurysms via fundus examination, thereby challenging the existing DR classification system.

The efficacy of UWF-directed OCT was assessed by Ashraf et al. Their pilot study revealed that this technique identified nearly 25.0% of suspected large IRMAs as NVE, some of which were not previously detected using UWF-C imaging (UWF-CI) [53]. These findings suggest that UWF-directed OCT could enhance PDR detection in eyes with advanced non-proliferative DR. However, larger studies are needed to compare its efficacy with existing imaging modalities.

### 2.5. Artificial Intelligence and Machine Learning in DR

Recent advances in AI and machine learning (ML), particularly through convolutional neural network (CNN) models, are revolutionizing ophthalmological diagnostics, especially in detecting and screening DR. AI, mimicking human cognitive functions, aids ophthalmologists in interpreting imaging and clinical data more effectively. Deep learning algorithms, including CNNs, excel in accuracy by learning from vast datasets. This integration promises more efficient and reliable DR screening methods, ultimately benefiting patients and healthcare providers [54,55].

In line with this transformative trend, several AI-powered tabletop devices have garnered FDA approval, showcasing remarkable performance in DR detection and classification. For instance, LumineticsCore™ formerly IDx-DR (Digital Diagnostics Inc., Coralville, IA, United States)achieved a sensitivity of 87.2% and a specificity of 90.7%, surpassing rigorous human grader standards as of 2023 [56]. Comparably, the EyeArt system automatically detects more-than-mild and vision-threatening DR, showing sensitivities of 94.6% and 92.7% and specificities of 85.9% and 92.4% for mtmDR and vision-threatening DR, respectively [57]. Finally, AEYE Diagnostic Screening (AEYE-DS) (AEYE Health, New York, NY, United States)targets mtmDR, demonstrating sensitivities of 94.7% and 93.0% and specificities of 88.6% and 91.4% with two and one image(s) per eye, respectively [58]. Additionally, as of today and according to the producers, it has been announced to be the first and only FDA-approved AI solution to allow autonomous screening anywhere using the Optomed Aurora portable handheld device, using only a single image from each eye [59]. Several other programs, including Retmarker (Retmarker, Coimbra, Portugal), Google (Google LLC, Mountain View, CA, United States), and Singapore Eye Lesion Analyzer (SELENA), hold class IIa approval (CE marking) in the EU, highlighting AI and ML’s potential in healthcare transformation and enhanced patient outcomes [60].

In a comprehensive review by Rajesh et al. (2023), AI systems demonstrated varying sensitivities and specificities for detecting DR. Sensitivities ranged from 83.3% (VoxelCloud Retina (VoxelCloud, Shanghai, China)) to a maximum of 96.9% (unnamed AI algorithm by Li et al.) while specificities varied between 85.0% (EyeArt) and 97.2% (SELENA for vision-threatening DR) [61]. Additionally, Wróblewski et al. tested two AI algorithms, Medios (offline) and EyeArt (online), using Remidio fundus-on-phone cameras. Medios achieved 94.0% sensitivity and specificity across all 248 patients, while EyeArt, influenced by photographer error, reached 94.0% sensitivity and 86.0% specificity across 156 patients [26]. Finally, Tang et al. developed a DL system, which showcased outstanding capabilities across various geographical regions in identifying referable DR (RDR) and vision-threatening DR (VTDR), achieving sensitivities of 94.9% and 87.2% and specificities of 95.1% and 95.8%, respectively [62]. These findings suggest promising prospects for remote, large-scale DR screenings, in some cases even without a constant internet connection.

Current research explores AI integration with various imaging modalities, including OCT, OCTA, FA, UWFI, and portable cameras, for comprehensive assessment of diabetic retinal pathology. Ryu et al. investigated a deep learning model for DR detection using OCTA, achieving accuracy ranging from 91.0% to 98.0%, sensitivity between 86.0% and 97.0%, and specificity from 94.0% to 99.0% [63]. Murata et al. developed an AI-inferred fluorescein angiography (AI-FA) system based on OCTA. The AI-FA closely resembled real FA images, achieving a high structural similarity index (0.9) with real FA images, accurately delineating vascular occlusion and associated leakage without the need for dye injection [64]. On the other hand, Silva et al.’s study demonstrated the effectiveness of automated ML models in identifying DR progression using UWF images [65]. 

Moreover, the introduction of UWF-Net, an advanced image enhancement algorithm, addresses issues in UWFI by improving the quality of ultra-widefield fundus images, which is crucial for detecting peripheral retinal pathologies often missed in traditional imaging. By enhancing UWFI images, UWF-Net significantly boosts AI performance in DR detection, offering a more reliable and comprehensive diagnostic tool that aligns better with the complexities of clinical practice [66].

The effective adoption of AI-based screening relies on patient trust and participation. Research evaluating patient adherence post-AI screening shows high satisfaction due to faster examinations and immediate results, leading to increased follow-up appointment attendance. For instance, Wolf et al.’s study on diabetic youth found that autonomous AI exams significantly increased screening completion rates, with 64.0% pursuing further consultations, compared to 22.0% in the control group [67]. Dow et al. also observed significantly higher ophthalmology follow-up rates after positive DR screening using AI Workflow, compared to Human Workflow or AI–Human Hybrid Workflow [68]. 

AI advancements in DR diagnosis and progression prediction allow for personalized follow-up intervals. For instance, DeepDR Plus, developed by Dai et al., extends the average DR screening interval from 12 months to nearly 3 years [69]. Rom et al. achieved an area under receiver operating curve (AUC) of 0.81 for multiple image scores, predicting over 3 years using machine learning on the EyePACS dataset [70]. Silva et al. demonstrated ML’s accuracy in predicting two-step or greater DR progression within 1 year [65]. These innovations reduce screening frequency, cutting treatment costs and enhancing efficiency. Moreover, they enable personalized treatment, ultimately improving patient outcomes. Economic studies suggest significant cost savings with AI integration into large-scale DR screening programs. Xie et al.’s research indicates a semi-automated approach, combining deep learning systems with human assessment, as the most financially beneficial option for large-scale screening programs [71].

Implementing AI and DL in DR diagnosis offers significant potential for enhancing screening efficiency and accuracy, but it comes with limitations. Reliance on large datasets for training may introduce biases if not representative of diverse populations. The performance of AI algorithms may degrade in real-world scenarios differing from training data [72,73]. Furthermore, the high initial costs associated with implementing AI technology, including infrastructure, training, and maintenance, may limit its accessibility [74]. Additionally, some AI programs may focus solely on one disease, such as DR, potentially overlooking others detectable through retinal imaging. For instance, IDx-DR is solely focused on DR, which means it can potentially overlook other ocular conditions; SELENA+, on the other hand, detects DR, as well as age-related macular degeneration and glaucoma [75]. Image quality remains a concern despite automated assessments, and cybersecurity risks must be addressed to protect patient data. Regulatory and ethical considerations require careful navigation for safe and effective healthcare delivery [76]. Addressing these challenges is crucial for realizing AI’s full potential in DR diagnosis while ensuring safe, effective, and equitable healthcare delivery.

### 2.6. Nanotechnology

**Figure 1:**
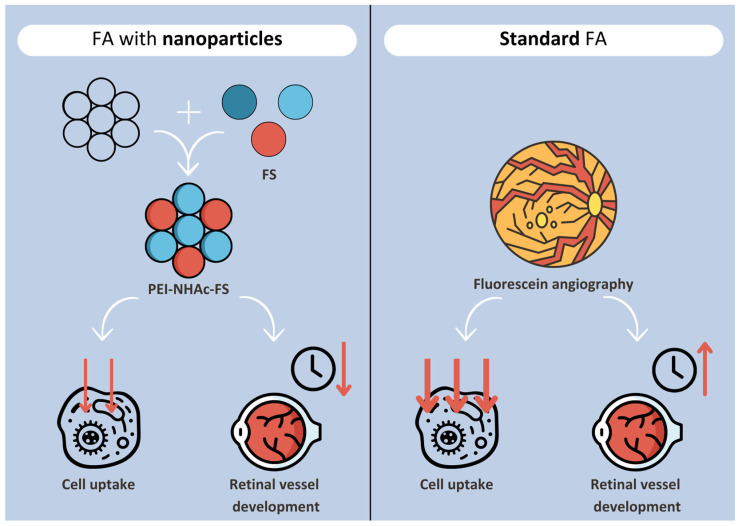
Schematic representation of PEI nanoparticle-aided FA in comparison with standard FA.

Nanotechnology has also been applied in fundus examination with fluorescein angiography, the gold standard screening in DR. Delivering intravenous fluorescein sodium (FS) through highly branched polyethyleneimine particles (PEI-NHAc-FS) reduces cellular uptake of the dye and its retention in the body, while maintaining optical performance comparable to free FS. Furthermore, it develops retinal vessels quicker, hence reducing the duration of fundus exposure to FS [77]. PEI-NHAc-FS are simple in preparation and have good biocompatibility and a high metabolic rate [78] (Figure 1).

Vedarethinam et al. proposed an advanced nanotechnology tool for metabolic fingerprints (MF) with vanadium oxide to distinguish DR patients from healthy controls with >90% sensitivity and specificity. With the use of silica nanorods, multiple vanadium core–shells are constructed, characterized by different compositions and structural parameters to optimize plasma MF through nanorod-assisted laser desorption/ionization mass spectrometry. Moreover, DR progression can be closely tracked through monitoring of gradual changes in plasma metabolic signatures [79].

**Figure 2:**
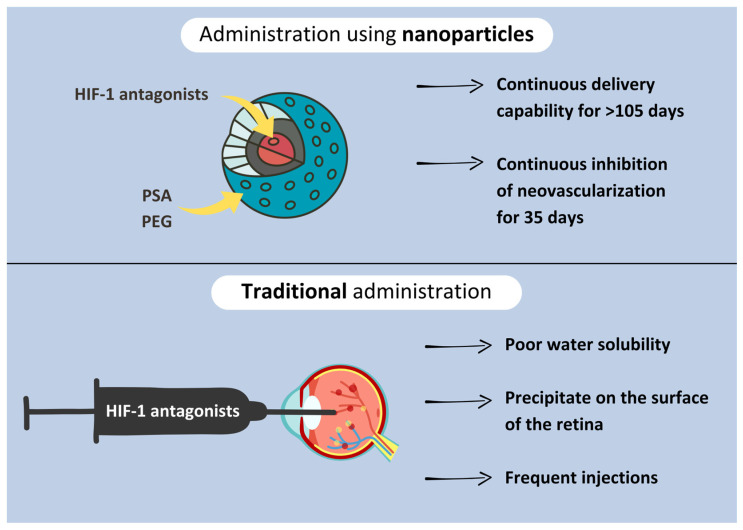
Figure comparing ways of HIF-1 antagonist administration.

Detecting and treating hypoxia before it induces retinal neovascularization is key to delaying the onset of DR. Antagonists of the hypoxia-inducible factor-1 (HIF-1), which promotes the expression of such growth factors like PDGF and VEGF have proven effective in preventing DR. Two of such inhibitors, doxorubicin and daunorubicin, traditionally administered through frequent intraocular injections, have poor water solubility and precipitate on the surface of the retina. Nanoparticles (NPs) using polysebacic acid (PSA) and polyethylene glycol (PEG) enable continuous delivery of daunorubicin to the retina, even for more than 105 days in some rabbit models with continuous inhibition of neovascularization for 35 days. The delivery system seems to be non-toxic to the retina; however, further investigations in humans are necessary [80] (Figure 2).

## 3. Conclusions

In conclusion, the modern approach to diagnosing DR integrates cutting-edge technologies such as teleophthalmology, widefield imaging, artificial intelligence, and nanotechnology. These innovations promise early detection and effective management of DR, potentially mitigating avoidable vision loss. FDA-approved AI systems demonstrate high sensitivity and specificity, improving screening accuracy. Tailored screening intervals guided by AI algorithms optimize resource utilization and enhance patient outcomes. However, addressing challenges like data biases, implementation costs, and data security is essential to fully harness the benefits of these advancements in DR diagnosis and management. Nanotechnology offers promising advancements in fundus examination and DR treatment by enhancing fluorescein delivery, distinguishing metabolic fingerprints, and enabling sustained drug release; however, further investigations in humans are necessary to ensure biocompatibility and safety. Despite these obstacles, the integration of modern technologies holds great potential in transforming the diagnosis and treatment of DR, ultimately elevating patient care and lessening the burden of diabetic eye disease.

## Figures and Tables

**Table 1:** Sensitivity and Specificity of Handheld Devices in Detecting Diabetic Retinopathy.

Authors & Year	Output Mydriatic (M)/Non-Mydriatic (NM)		Sensitivity/Specificity (%)	Device (with AI System)
Han et al., 2021 [19]	M	Referable Diabetic Retinopathy (DR)	95.0/98.0	Paxos Scope (Verana Health, San Francisco, CA, USA)
Toy et al., 2016 [21]	M		91.0/99.0	
Russo et al., 2015 [22]	M	Any DR	86.0/96.0	D-EYE (Si14 S.p.A., Padova, Italy)
		Diabetic Macular Oedema (DME)	81.0/98.0	
Sengupta et al., 2019 [23] Rajalakshmi et al., 2015 [24]	M	Any DR	93.7/91.8	Remidio Fundus on Phone FOP (Remidio Innovative Solutions, Bangalore, India)
			92.7/98.4	
Rajalakshmi et al., 2018 [25]	M	Any DR	95.8/80.2	Remidio FOP withEyeArt online(Eyenuk, Inc., Los Angeles, CA, USA)
		Vision-threatening DR	99.1/80.4	
Wroblewski et al., 2023 [26]	M	Any DR	94.0/86.0	Remidio FOP with EyeArtonline
	M	Any DR	94.0/94.0	Remidio FOP with Medios offline(Medios Technologies, Singapore)
Kim et al., 2018 [27]	M	Referable DR	93.3/56.8	CellScope Retina (not mentioned in the study)
		DME	49.1/90.7	
Salongcay et al., 2022 [28]	NM	Any DR	79.0/97.0	Optomed Aurora (Optomed Ltd, Oulu, Finland)
		DME	65.0/100.0	
		Any DR	86.0/97.0	
	M	DME	80.0/99.0	
Midena et al., 2022 [29]	M	Any DR	96.9/94.8	Optomed Aurora
		DME	100.0/99.8	
Zhou et al., 2024 [16]	NM	Any DR	90.9/100.0	Optomed Aurora
Lupidi et al., 2023 [30]	M	Any DR	96.8/96.8	Optomed Aurora; with Selena + (EyRIS, Pte Ltd. Singapore)
Doğan et al., 2024 [31]	NM	Vision-threatening DR	95.12/98.2	Optomed Aurora; (EyeCheckup) (Ural Telecommunication Inc., Akdeniz University Teknokent, Antalya)
Ruan et al., 2022 [32]		Referable DR	88.2/40.7	Optomed Aurora (Phoebus, Shanghai, China)
Salongcay et al., 2022 [28]	NM	Any DR	80.0/96.0	SmartScope (Optomed Ltd, Oulu, Finland)
		DME	72.0/100.0	
		Any DR	80.0/92.0	
	M	DME	75.0/100.0	
Salongcay et al., 2022 [28]	NM	Any DR	89.0/88.0	RetinaVue-700 (Welch Allyn, Skaneateles Falls, NY, United States)
		DME	76.0/99.0	
		Any DR	83.0/97.0	
	M	DME	87.0/98.0	
Salongcay et al., 2022 [28]	M	Any DR	91.0/53.0	iNview (Volk Optical Inc, Mentor, OH, United States)
		DME:	ungradable rate	
de Oliveira et al., 2023 [15]	M	Vision-threatening DR	90.6/80.8	The Eyer
			87.3/82.7	
Nunez do Rio et al., 2022 [33]	NM	Referable DR	72.08/85.65	Zeiss Visuscout with VISUHEALTH-AI DR (Software version 1.8) (Carl Zeiss Meditec, Jena, Germany)
Rajalakshmi et al., 2024 [17]	M	Vision-threatening DR	92.7/96.6	Remidio Vistaro

**Table 2:** Summary of Key Findings from Studies on WF and UWF Imaging.

Study	Imaging Method	Key Findings
Silva et al., 2015 [37]	Optos P200MA (Optos plc, Dunfermline, UK)	Eyes with predominant peripheral lesions (PPLs) had a 3.2-fold higher risk of worsening diabetic retinopathy (DR) and a 4.7-fold higher risk of progressing to proliferative diabetic retinopathy (PDR). Around 50% of the eyes in this study had DR lesions primarily situated outside the coverage of the standard Early Treatment Diabetic Retinopathy Study (ETDRS) (7F) photographic view.
Duncan et al., 2024 [35]	Zeiss Clarus, Optos Colour UWF	Strong agreement with 7F for DR detection and staging; fewer ungradable images with Clarus (2.1%) and Optos (1.0%) than with 7F (5.2%). UWF imaging detects proliferative lesions in the peripheral retina missed by 7F. Higher costs and potential artifacts from eyelashes, eyelid margins, and distortion. UWF systems identified more PDR cases outside the 7F area: Clarus (5.6%) and Optos (8.2%). Clarus had 85% exact agreement vs. 76% for Optos.
Talks et al., 2015 [41]	WFI Optos Optomap P2000	Detected 11.7% more PDR cases and 30% more NVs than standard imaging.
Wessel et al., 2012 [40]	UWFA using Optos Optomap Panoramic 200A imaging system	Detected retinal neovascularization in 17% of PDR cases missed by standard seven-field imaging.
Rajalakshmi et al., 2024 [17]	UWF Optos Daytona Plus (Optos Inc, Marlborough, MA, USA)	Widefield imaging (UWF) detects peripheral lesions outside the traditional seven fields without dilation. Optos Daytona Plus identified peripheral DR lesions in over 50% of eyes, primarily in the supero-temporal quadrant. The sensitivity for detecting proliferative diabetic retinopathy (PDR) is 73% to 80%, with a high specificity of 96% to 99.3%. Challenges include color variations, pseudocolor artifacts, and peripheral image distortion, which can complicate the identification of lesions like intraretinal microvascular abnormalities (IRMAs).
Srinivasan et al., 2023 [42]	UWF Digital Imaging Daytona Plus	A 15.2% grading disagreement due to low resolution for small lesions like microaneurysms. Discrepancies in differentiating DR severity, especially between no DR and mild DR, and moderate vs. severe non-proliferative DR (NPDR). Optos UWF has lower resolution and pseudocolor imaging issues, affecting small lesion detection and grading. UWF captures a wider field but may have focus issues in peripheral lesions, sometimes showing higher DR severity than the ETDRS seven-field view.

## Data Availability

No new data were created or analyzed in this study. Data sharing is not applicable to this article.

## Abbreviations

DM	Diabetes mellitus
DR	Diabetic retinopathy
PDR	Proliferative diabetic retinopathy
DME	Diabetic macular edema
SBP	Smartphone-based photography
AI	Artificial intelligence
DL	Deep learning
CFP	Color fundus photography
FFA	Fundus fluorescein angiography
OCT	Optical coherence tomography
OCTA	OCT angiography
WFP	Widefield photography
SLB	Slit-lamp biomicroscopy
SBFP	Smartphone-based fundus photography
UWFI	Ultra-widefield imaging
WFI	Widefield imaging
mtmDR	More-than-mild DR
UWF-C	Ultra-widefield Colour
UWF	Ultra-widefield
FA	Fluorescein angiography
FOV	Field of view
NV	Retinal neovascularization
WF-OCTA	Widefield OCTA
NVE	NV elsewhere
SS-OCTA	Swept-source OCTA
RGB	Red–Green–Blue
RG	RedºGreen
PPLs	Predominant peripheral lesions
ETDRS	Early Treatment Diabetic Retinopathy Study
NPDR	Non-proliferative DR
ML	Machine learning
IRMAs	Intraretinal microvascular abnormalities
UWF-SS-OCTA	Ultra-widefield swept-source OCTA
DM-NoDR	Diabetic patients without clinical DR
NPA	Non-perfusion areas
UWF-CI	UWF-C imaging
CNN	Convolutional neural network
AEYE-DS	AEYE diagnostic screening
RDR	Referable DR
VTDR	Vision-threatening DR
AUC	Area-under-receiver operating curve
PEI-NHAc-FS	Polyethyleneimine particles with fluorescein sodium
FS	Fluorescein sodium
MF	Metabolic fingerprints
HIF-1	Hypoxia-inducible factor-1
NPs	Nanoparticles
PSA	Polysebacic acid
PEG	Polyethylene glycol
